# An infographic on laboratory animal veterinarians

**DOI:** 10.1186/s42826-025-00244-8

**Published:** 2025-09-08

**Authors:** Ji-Young Kim, Young-Shin Joo, Yujin Kim, On Shim, Jae-Hun Ahn, Jae-eun Lee, Jiwon Lee, Jinha Jeon, Da In On, Yu Gang Kim, Bora Kim, Seung-Yeon Kim, Insook Yang, Kyoung-Sun Lee, Jungmin Lee, Ji-Yeon Hwang, Hyunjhung Jhun, Jun-Won Yun, Jeong-Hwan Che, Byeong-Cheol Kang, Ki Taek Nam, Seung Hyun Oh, Je Kyung Seong

**Affiliations:** 1 College of Medicine, Ewha Woman University, Seoul, Republic of Korea; 2 Laboratory Animal Research Center, Institute of Biomedical Industry, The Catholic University of Korea, Seoul, Republic of Korea; 3 Yonsei Laboratory Animal Research Center, Yonsei University, Seoul, Republic of Korea; 4 Department of Experimental Animal Research, Biomedical Research Institute, Seoul National University Hospital, Seoul, Republic of Korea; 5 Laboratory Animal Research Center, Sungkyunkwan University, Seoul, Republic of Korea; 6 Preclinical Research Center, Biomedical Research Institute, Seoul National University Bundang Hospital, Seongnam, Republic of Korea; 7 Korea Model Animal Priority Center (KMPC), Seoul, Republic of Korea; 8 Research Core Center, Research Institute, National Cancer Center, Goyang, Republic of Korea; 9 Animal Research Facility, International Vaccine Institute, Seoul, Republic of Korea; 10 Department of Laboratory Animal Resources, Biomedical Research Institute, Yonsei University College of Medicine of Korea, Seoul, Republic of Korea; 11 Non-Clinical Evaluation Center, OSONG Medical Innovation Foundation (K BIO : Korea Bio-cluster), Cheongju, Republic of Korea; 12 Preclinical Resource Center, Samsung Medical Center, Seoul, Republic of Korea; 13 Infrastructure Support Team, Korea Food Research Institute, Wanju, Republic of Korea; 14 Laboratory of Veterinary Toxicology, Research Institute for Veterinary Science, College of Veterinary Medicine, Seoul National University, Seoul, Republic of Korea; 15 BK21 FOUR Future Veterinary Medicine Leading Education and Research Center, College of Veterinary Medicine, Seoul National University, Seoul, Republic of Korea; 16 Biomedical Center for Animal Research Development, Seoul National University College of Medicine, Seoul, Republic of Korea; 17 Seoul National University College of Medicine, Seoul, Republic of Korea; 18 Department of Biomedical Sciences, Yonsei University College of Medicine, Seoul, Republic of Korea; 19 Laboratory of Veterinary Histology, Research Institute for Veterinary Science, College of Veterinary Medicine, Seoul National University, Seoul, Republic of Korea; 20 Laboratory of Developmental Biology and Genomics, Research Institute for Veterinary Science, College of Veterinary Medicine, Seoul National University, Seoul, Republic of Korea

**Keywords:** Laboratory animal, Laboratory animal veterinarian, Attending veterinarian, Veterinary care, Infographics, Korean College of Laboratory Animal Medicine (KCLAM), Diplomate of the Korean College of Laboratory Animal Medicine (DKCLAM)

## Abstract

**Background:**

Laboratory animal veterinarians play a crucial role as a bridge between the ethical use of laboratory animals and the advancement of scientific and medical knowledge in biomedical research. They alleviate pain and reduce distress through veterinary care of laboratory animals. Additionally, they enhance animal welfare by creating environments that mimic natural habitats through environmental enrichment and social associations. This approach reduces errors caused by improper animal management and enhances the reproducibility of animal experiments, thereby contributing significantly to scientific progress.

**Results:**

The Korean College of Laboratory Animal Medicine, established in 2006, aims to formalize the status of laboratory animal veterinarians. The revised Animal Protection Act of April 2022 mandates the employment of attending veterinarians in animal research facilities exceeding prescribed standards by Presidential Decree. This underscores the increasing importance of laboratory animal veterinarians in Korean society. Consequently, the Korean College of Laboratory Animal Medicine initiated efforts to raise awareness of laboratory animal veterinarians, leading to the creation of an infographic. Infographics combine textual and graphical elements to effectively convey information, data, and knowledge. These veterinarians collaborated with infographic specialists to research, check, classify, refine, analyze, and structure content on laboratory animal veterinarians.

**Conclusion:**

This infographic represents the first comprehensive initiative worldwide on laboratory animal veterinarians. It will be disseminated globally to animal research facilities to enhance awareness and promote the professional standing of laboratory animal veterinarians.

## Background

The use of animal models in preclinical and basic biomedical research is increasing [1, 2]. Animal research institutions have more than tripled, and laboratory animal usage has increased 6.5-fold from 2008 to 2022 in Korea. Recent reports indicate that there are 517 animal institutions, utilizing > 4.99 million laboratory animals such as rodents, rabbits, non-human primates, birds, and fish for scientific purposes [3].

Laboratory animal veterinarians (LAVs) work to care for and improve the health and welfare of laboratory animals used in research, testing, and education. They play an indispensable role in the advancement of medical life sciences and healthcare by providing veterinary care to laboratory animals through daily observation, overseeing environment and husbandry to enhance health and welfare of laboratory animals, managing animal facilities, providing guidance to researchers and staff, and training and assisting these people using medical and scientific knowledge [4].

Animal research facilities that meet the specified criteria are required to employ attending veterinarians (AV) who are responsible for the health, welfare, ethical use, and veterinary care of laboratory animals, following the full implementation of the revised Animal Protection Act in 2023 in Korea. The Korean College of Laboratory Animal Medicine (KCLAM) developed this infographic to advocate for and highlight the essential role of LAVs.

## Main text

An infographic is a visual representation comprising information and graphics designed to effectively communicate complex concepts to the public using images or figures. It serves as a pictorial demonstration of data or knowledge aimed at clearly and quickly disseminating information through social media platforms in the digital era [5].

This infographic on LAVs is structured into three main sections. The first section introduces LAVs, providing an overview of their profession and significance. The second section details the roles and responsibilities of LAVs. Lastly, the infographic summarizes the key terms used.

### Steps for creating an infographic

**Fig. 1:**
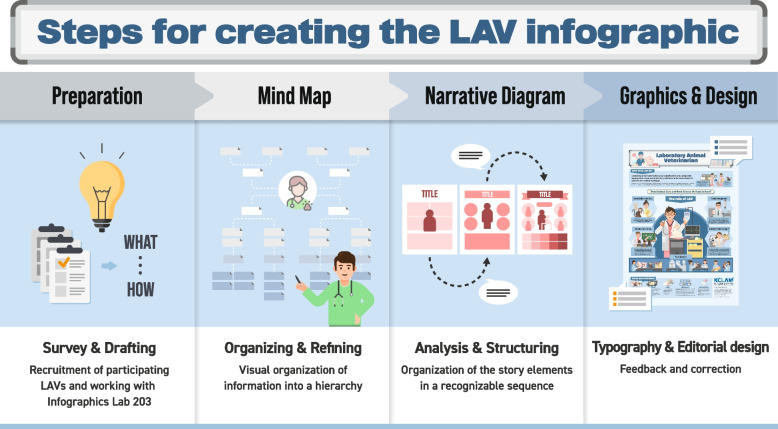
Steps for creating the LAV infographic. The process of creating the LAV infographic included four steps: preparation, mind mapping, narrative diagrams, and graphics and design. Preparation: The KCLAM recruited volunteer participants, including both attending and entry-level veterinarians. The infographic team also collaborated with mentors who had expertise in laboratory animal medicine and a graphic design agency (Lab 203). During a brainstorming meeting, we defined the overarching goals and messages for publicizing LAVs. Mind mapping: We collected data and created a hierarchy to describe the definition and role of LAVs. Narrative diagram: To visualize the data, we used an infographic template and organized the information for each topic. Graphics and design: We added and revised the detailed designs of the infographic based on feedback from our mentors. This infographic is designed to inform the public about LAVs in an easy-to-read format. LAV, laboratory animal veterinarian; KCLAM, Korean College of Laboratory Animal Medicine

To create this infographic on LAVs, KCLAM assembled an infographics production team. The team comprised 16 LAVs and six professors who have served as AVs or specialists in laboratory animal facilities affiliated with universities, hospitals, public institutions, and research institutions. They possess diverse experiences and expertise in laboratory animals and their use in research. Additionally, we collaborated with an infographics specialist (Infographics Lab 203) to develop an outline of LAVs. This involved conducting research, categorizing and refining data (Mind map), and analyzing and structuring content (Graphic and Design). Following the themes of “LAV” (What), “LAVs” (By Who), we created a poster (How) aimed at informing researchers and the general public (For Whom) about the roles and importance of LAVs (Why). This process entailed data refinement post-research, the establishment of a relationship map for LAVs, and the presentation of information visually using shape and text elements (Fig. 1).

### What do LAVs do?

A fundamental question often arises regarding the involvement of LAVs in biomedical research. LAVs, equipped with medical knowledge, bear the responsibility for the ethical and legal oversight of veterinary care and the use of laboratory animals [6, 7]. These professionals, alternatively referred to as AVs or Diplomates of the Korean College of Laboratory Animal Medicine (DKCLAM), undergo certification, possess extensive professional experience, and have completed the mandatory education in their field.

### What are the roles of LAVs?

**Fig. 2:**
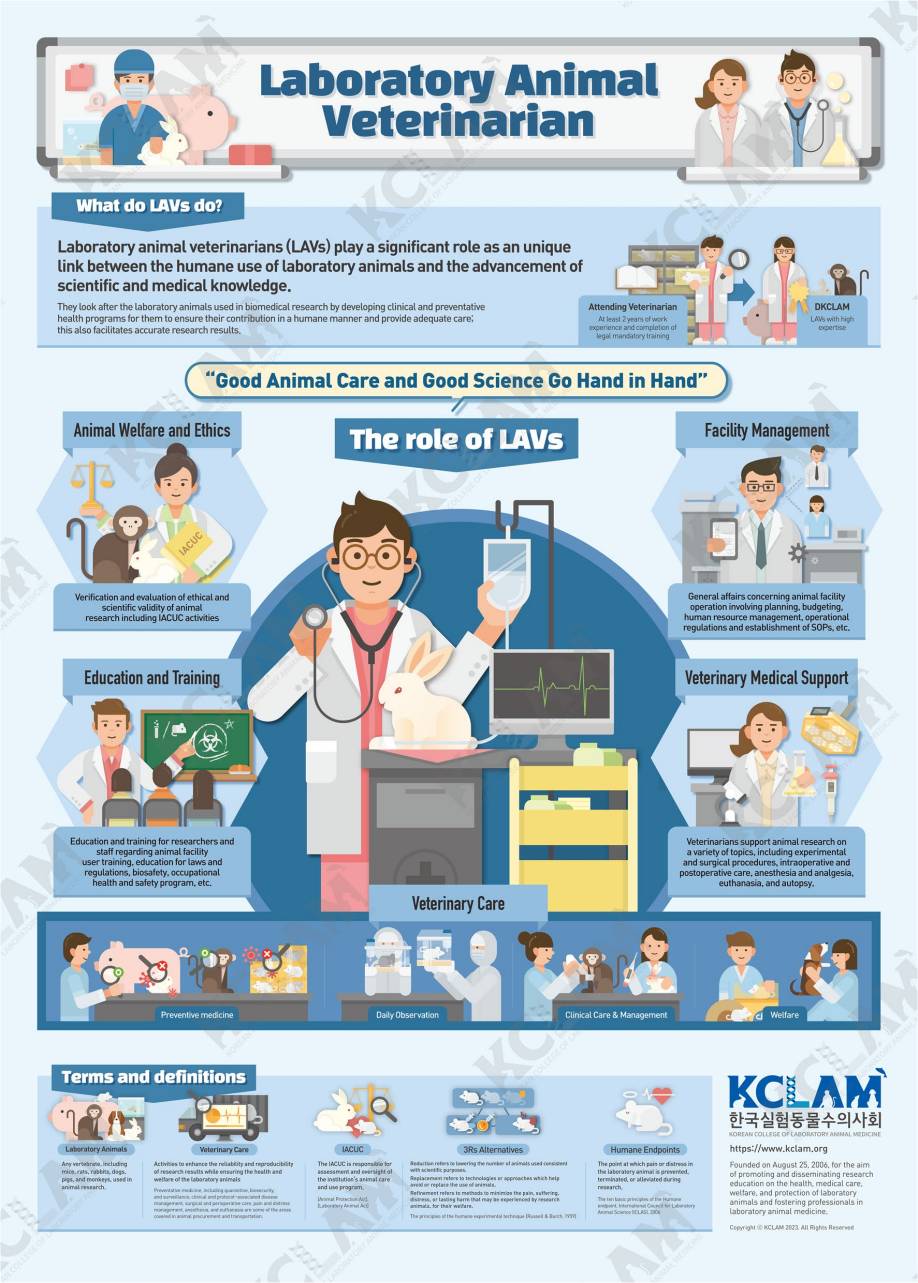
Laboratory animal veterinarian infographic. The LAV infographic created by the KCLAM provides accurate information about veterinarians in biomedical research to inform the public. LAVs perform various roles to achieve scientific and ethical goals in research involving animals. They are responsible for providing high-quality animal care and ensuring compliance with regulations. Their duties include—good veterinary care: preventive medicine, daily observation, clinical care and management, and overall welfare; animal welfare and ethics: involvement in IACUC activities; education and training: providing training on laws and regulations, biosafety, and occupational health and safety programs; facility management: handling planning, budgeting, human resource management, establishing standard operating procedures; and veterinary medical support: assisting with surgery, intraoperative and postoperative care, anesthesia and analgesia, euthanasia, and autopsy. LAV, laboratory animal veterinarian; KCLAM, Korean College of Laboratory Animal Medicine; IACUC, Institutional Animal Care and Use Committee. “Good Animal Care and Good Science Go Hand in Hand [9]”

LAVs are versatile experts in animal welfare and possess scientific knowledge of animal facilities [8]. The roles and responsibilities of LAVs must include the following categories: (1) veterinary care, including preventative medicine, daily observation, clinical care, management and welfare; (2) animal welfare and ethics, involving the verification and evaluation of ethical and scientific validity of animal research, including Institutional Animal Care and Use Committee (IACUC) activities; (3) education and training for researchers and staff regarding animal facility user training, laws and regulations, biosafety, occupational health, and safety programs; (4) facility management, encompassing general affairs related to animal facility operation, including planning, budgeting, human resource management, operational regulations, and establishment of standard operating procedures; (5) veterinary medical support for animal research, covering various topics such as experimental and surgical procedures, intraoperative and postoperative care, anesthesia and analgesia, euthanasia, and autopsy (Fig. 2).

### Terms and definitions

A brief explanation of the terminology used in animal research and by LAVs, such as laboratory animals, veterinary care, IACUC, 3R alternatives, and humane endpoints, is provided at the bottom of this infographic for clarity.

## Conclusions

The first full amendment to the Animal Protection Act in Korea, as implemented in 2008. This law mandated the establishment of an IACUC for ethical animal research and required annual reports on the number of animals used in research for all animal facilities. A comprehensive revision of the law took place in 2022. According to the amended law, animal facilities exceeding the standards prescribed by Presidential Decree (No. 33435) are obligated to hire certified LAVs, such as AVs exclusively responsible for laboratory animals. These regulations were initially established in Asia. In contrast, in the United States, AVs are specified by the Animal Protection Act and Health Research Extension Act, and the European Union mandates designated veterinarians in animal research facilities through DIRECTIVE 2010/63/EU, requiring the hiring of LAVs.

Recent reports have demonstrated that good animal care is crucial for research reliability [10, 11]. LAVs play a critical role in overseeing and safeguarding animal care, using programs to ensure animal health and welfare. Therefore, the veterinary services provided by well-trained LAVs are essential for high-quality science.

Despite the significance, there has been a scarcity of intuitive resources that are easy to use to promote awareness of LAV and foster social understanding. More than 20 experts in KCLAM created this LAV infographic to concisely describe who LAVs are, what they do, and the commonly used terminology in this field. Through this LAV infographic, we aim to convey the essential role of LAVs in caring for laboratory animals and ensuring scientifically and ethically conducted experiments. We hope this will contribute to enhancing public awareness and serve as a tool to raise awareness regarding LAVs, not only in Korea but also across Asia and other countries.

## Data Availability

All relevant results are presented herein. The datasets used and/or analyzed in the current study are available from the corresponding author upon reasonable request.

## Abbreviations

LAVs: Laboratory animal veterinarians
IACUC: Institutional Animal Care and Use Committee
APA: Animal Protection Act
AV: Attending veterinarian
KCLAM: Korean College of Laboratory Animal Medicine
DKCLAM: Diplomate of the Korean College of Laboratory Animal Medicine
